# Severity of Premenstrual Symptoms Predicted by Second to Fourth Digit Ratio

**DOI:** 10.3389/fmed.2017.00144

**Published:** 2017-09-04

**Authors:** Yoshiki Kaneoke, Tomohiro Donishi, Akihiko Iwahara, Toshio Shimokawa

**Affiliations:** ^1^Department of System Neurophysiology, Wakayama Medical University, Wakayama, Japan; ^2^School of Health and Nursing Science, Wakayama Medical University, Wakayama, Japan; ^3^Clinical Research Center, Wakayama Medical University, Wakayama, Japan

**Keywords:** 2D:4D, prenatal sex hormones, testosterone, estrogen, reproductive age

## Abstract

Women of reproductive age often experience a variety of unpleasant symptoms prior to the onset of menstruation. While genetics may influence the variability of these symptoms and their severity among women, the exact causes remain unknown. We hypothesized that symptom variability originates from differences in the embryonic environment and thus development caused by variation in exposure to sex hormones. We measured the second to fourth digit ratios (2D:4D) in 402 young women and investigated the potential relationships of this ratio premenstrual symptoms using a generalized linear model. We found that two models (one with two predictors such as both hands’ digit ratios and the other with the difference between the two digit ratios, Dr-l) were significantly different from the constant model as assessed by chi-square test. The right digit ratio and Dr-l were negatively related to the symptom scores, and the left digit ratio was related to the scores. When premenstrual symptoms were classified into eight categories, five categories, including pain, concentration, autonomic reaction, negative affect, and control were associated with the digit ratios and Dr-l. Behavioral changes and water retention were not predicted by them. Arousal was predicted by Dr-l. The right 2D:4D is thought to be determined by the balance of testosterone and estrogen levels during early embryogenesis and is not affected by postpartum levels of sex hormones, while the left 2D:4D might be affected by the other prenatal environmental factors. We conclude that the embryonic environment, including the relative concentration of sex hormones an embryo is exposed to, is associated with the severity of premenstrual symptoms once menarche is reached.

## Introduction

Up to 90% of women of reproductive age experience discomfort related to their menstrual cycle, with 20–40% of women manifesting severe symptoms that meet the criteria for “premenstrual syndrome” (PMS) (1). Although a number of studies involving families and twins have supported the hypothesis that premenstrual symptoms are under genetic influence (2, 3), the precise genes and environmental stressors involved remain largely unknown. Severity and symptoms considerably vary among women. Some women develop central nervous system complaints and others develop symptoms in their peripheral organs that exhibit sensitivity to fluctuations in hormonal status and cause a variety of symptoms in response to environmental stress. Paradoxically, the magnitude of hormonal level changes cannot explain the variability (4), even though the symptoms occur with the menstrual cycle.

One possibility is that the embryonic environment, particularly exposure to different levels of sex hormones, may determine individual sensitivity to fluctuation of sex hormones in women of reproductive age by the so-called organizational effects (5). Sex hormones are known to influence both brain function (6–9) and development (10) and a number of studies have shown that exposure to prenatal sex hormones can affect brain development, cognitive skills, and a number of behavioral traits in adulthood (11–14). Although prenatal sex hormone levels do not correlate with adult sex hormone levels (15), they are associated with the magnitude of increase with challenging situations in adults (16). We hypothesized that if premenstrual symptoms originate from the development of the brain and other organs that are thought to cause vulnerability to fluctuations in sex hormones in females, then exposure to prenatal sex hormones might affect their severity.

To address this hypothesis, we used the second to fourth digit ratios (2D:4D) to estimate prenatal sex hormone exposure levels. Manning et al. were the first to propose that exposure to prenatal sex hormones might exert an effect on the 2D:4D (17). Other studies have supported Manning’s viewpoint (18, 19), and the causal relationship between testosterone and estrogen to digit ratio has also been reported in animal experiments (20). Testosterone increases the 4D length, which results in a smaller digit ratio, while estrogen reduces the 4D length and causes an increase in digit ratio. Consequently, female digit ratio is generally higher than that of males (21). Although a number of studies have supported that 2D:4D is a biomarker for the balance between fetal testosterone and estrogen, the digit ratio is also under genetic control (22, 23), which might be related to the prenatal sex hormones levels; however, other genes might directly affect digit ratios independent of sex hormones. Thus, the digit ratio is associated with the prenatal sex hormones but it must also be affected by the other factors, which leads to the idea that a substantial amount of data is required to elucidate the effects of prenatal sex hormones using the digit ratios.

In this study, we investigated the relationship between both hands’ digit ratios and premenstrual symptoms using 402 young university student females. If any of these symptoms were shown to be linked to digit ratio, then this would represent the first evidence that exposure to prenatal sex hormones are associated with premenstrual symptoms in women of reproductive age.

## Materials and Methods

This study was carried out in accordance with the Declaration of Helsinki and was approved by the Ethics Committee of Wakayama Medical University. Written informed consent was obtained from all subjects. All 403 participants were female students in the Universities in Wakayama City and had no health problems in attending classes. They saw posters that described the objective of this study and advertised for recruitment of subjects, and then independently decided to participate in the study. They received a coupon for 500 yen (about 4.50 US dollars) as a reward. The mean age was 19.6 ± 1.6 years old (mean ± SD).

We used a “Menstrual Distress Questionnaire” (MDQ) developed by Moos (24) to investigate the premenstrual symptoms of each subject because the Japanese version of this particular questionnaire has been commonly used in Japan. The questionnaire assesses 47 symptoms and subjective severity is rated as none, weak, mild, and severe. For statistical analysis, we referred to these subjective responses as 0, 1, 2, or 3, respectively. First, we evaluated the relationship between the total score (sum of the scores of all 47 symptoms) and digit ratios (see below). Second, 46 of the symptoms were used to score eight specific categories: pain, concentration, behavioral change, autonomic reactions, water retention, negative affect, arousal, and control as suggested by Moos (24). The score for individual subjects was determined by the sum of the numbers given by each participant in each symptom category: “Pain” included six symptoms: muscle stiffness, headache, cramps, backache, fatigue, and general aches and pains. “Concentration” included eight symptoms: insomnia, forgetfulness, confusion, lowered judgment, difficulty concentrating, distractible, accidents, and lowered motor coordination. “Behavioral change” included five symptoms: lowered school or work performance, take naps (or stay in bed), stay at home, avoid social activities, and decreased efficiency. “Autonomic reactions” included four symptoms: dizziness (or faintness), cold sweats, nausea (or vomiting), and hot flashes. “Water retention” included four symptoms: weight gain, skin disorders, painful breasts, and swelling. “Negative affect” included eight symptoms: crying, loneliness, anxiety, restlessness, irritability, mood swings, depression, and tension. “Arousal” included five symptoms: affectionate, orderliness, excitement, feeling of well-being, and bursts of energy (or activity). “Control” included six symptoms: feeling of suffocation, chest pains, ringing in the ears, heart pounding, numbness (or tingling), and blind spots (or fuzzy vision).

For each subject, we scanned the image of both hands using a portable color scanner (CanoScan Lide 210, Canon Inc.) with a spatial resolution of 4,800 × 4,800 dpi. These images were used to measure digit length. The second (2D) and fourth digit (4D) length in both hands were measured from the proximal finger crease to the distal tip of the finger, as described in a previous study (17). Three to four examiners used digital Vernier calipers to the nearest 0.01 mm, and the mean value was used to calculate digit ratio (2D:4D). The data from one subject were excluded from analysis because the proximal finger crease of the forth digit could not be detected. Therefore, we used 402 subjects’ data for the analyses. For each hand, the 2D length divided by the 4D length was used to obtain the digit ratio (2D:4D), and the difference between right and left 2D:4D (right value minus left value) was used to calculate the difference, Dr-l.

### Statistical Analysis

Pearson’s correlation coefficient was used to assess the relationship between right and left hand 2D:4D values. When there was a statistically significant correlation between the digit ratios, we calculated the variance inflation factor (VIF) (25) to evaluate for the effect of multicollinearity in the multivariate analysis. Next, we used a generalized linear model (GLM) to assess the relationship between digit ratios and premenstrual symptoms. We chose a Poisson distribution model with a log link function, because the symptom scores are natural numbers and the distributions of the scores could follow a Poisson distribution (see below) as used in the several previous studies (26–30). The digit ratios of both hands were included as predictor variables because each value could be affected by prenatal sex hormones in different ways (17, 18, 22, 31). Next, we assessed if Dr-l alone could predict the symptom scores using the same GLM analysis. Low Dr-l is related to the sensitivity of androgen receptors as right 2D:4D (32), which are known to influence various human traits (16). We also assessed one-predictor models using the right and left digit ratios alone to compare the results with two-predictor models using both the right and left digit ratios. The Akaike’s information criterion (AIC) was used to compare goodness of fit among the models (33).

## Results

**Figure 1:**
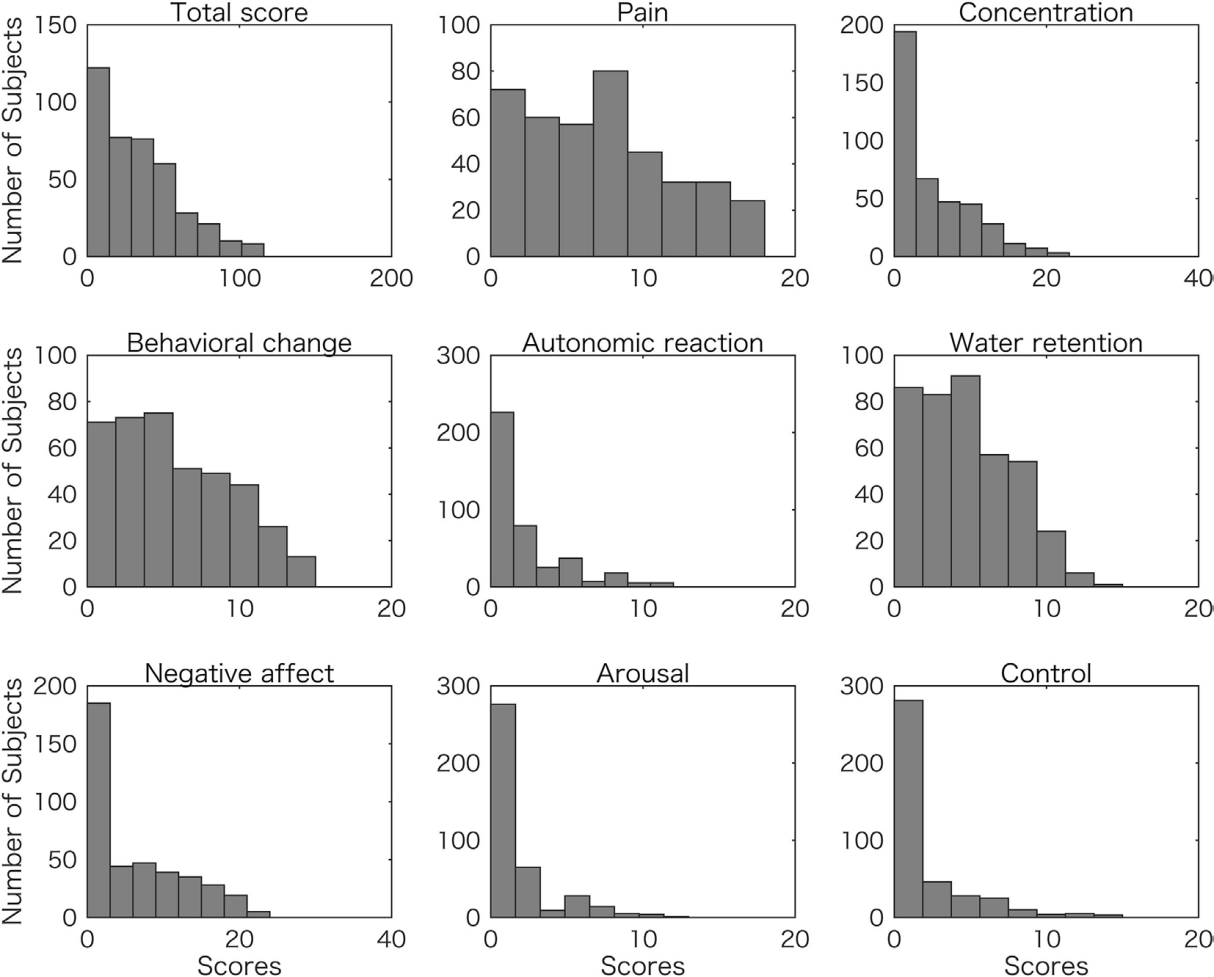
Frequency distributions of student scores for overall (total) and eight symptom scores. The number of subjects are plotted with total premenstrual symptom scores (Total score), and each of the symptom scores of pain, concentration, behavioral change, autonomic reaction, water retention, negative affect, arousal, and control.

The mean digit ratio 2D:4D for the right hand was 0.963 ± 0.026 and for the left hand was 0.966 ± 0.026 (mean ± SD). The right-hand value was significantly correlated with the left-hand’s value (*r* = 0.567, *p* = 1.42 × 10^−35^), and the VIF was 1.47. The mean (±SD) of Dr-l was −0.003 (±0.024). Figure 1 shows the frequency (number of subjects) and distribution of premenstrual symptom scores (total score and scores of the eight categories) (see Materials and Methods). The distributions were not symmetrical, with frequency decreasing as the score of premenstrual symptom score increased.

**Figure 2:**
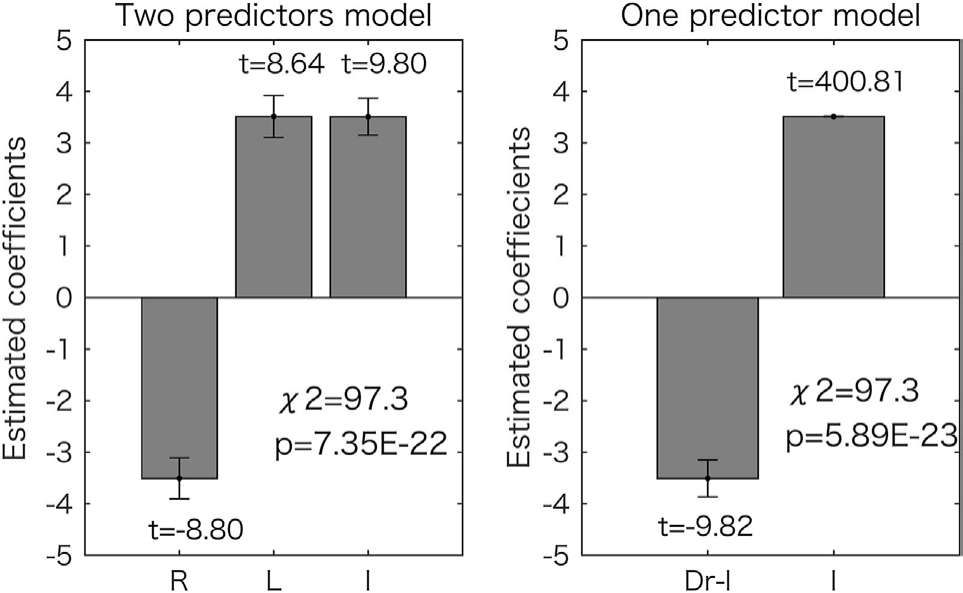
Generalized linear model analyses for the overall (total) scores. Results for the two-predictor model (left side) and the one-predictor model (right side) are shown. The estimated coefficient values (±SE) are shown with *t* values (all the values are *p* < 0.05). The chi-square tests revealed that both models were significantly different from the constant model as shown in each graph. R, right 2D:4D; L, left 2D:4D; I, intercept.

When total scores were estimated by the digit ratios using a GLM (see Materials and Methods), the model using two predictors, including right and left 2D:4D, could predict the scores with significantly increased accuracy as compared to the constant model, according to the chi-square test (df = 399, *p* = 7.35 × 10^−22^). The other model, using Dr-l as a predictor, also produced significantly improved results as compared to the constant model (df = 400, *p* = 5.89 × 10^−23^). The estimated coefficients and *t* values are shown in Figure 2. All the values were significantly different from zero (*p* < 1.0 × 10^−17^). According to the model with two predictors, the right 2D:4D was negatively related to the score and the left 2D:4D was positively related to the score. One-predictor model showed that Dr-l was negatively related to the score.

**Figure 3:**
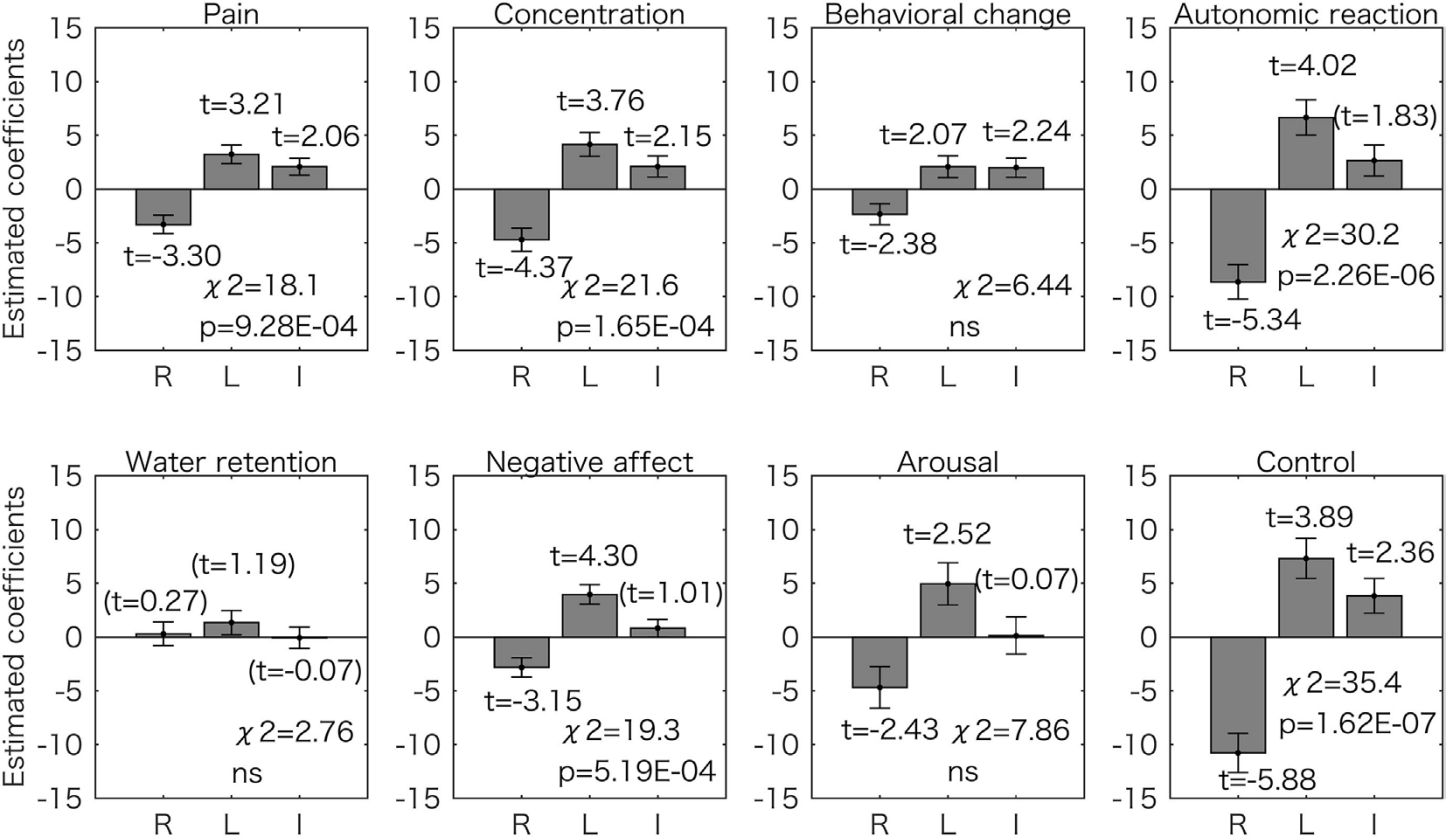
Generalized linear model analyses using two predictors for the 8 symptom scores. Estimated coefficient values (±SE) are shown with *t* values. All the *t* values (except the values in the parentheses) are *p* < 0.05. The *p* values for the chi-square test are corrected for multiple comparisons using the Bonferroni method. For five symptom scores (pain, concentration, autonomic reaction, negative affect, and control), the right 2D:4D was negatively associated to symptom scores and the left 2D:4D was positively associated. ns, not significant (*p* > 0.05); R, right 2D:4D; L, left 2D:4D; I, intercept.

Next we assessed if each symptom score (see Materials and Methods) could also be estimated by the two models. The results for the model using the right and left 2D:4D as predictors are shown in Figure 3. Most of the estimated coefficients are significantly different from zero (*p* < 0.05), and five symptom scores (pain, concentration, autonomic reaction, negative affect, and control) could be predicted by the two values in a similar way (*p* < 0.05 corrected for multiple comparisons using the Bonferroni method by chi-square test). The right 2D:4D was negatively related to the score, and the left 2D:4D was positively related to the score.

**Figure 4:**
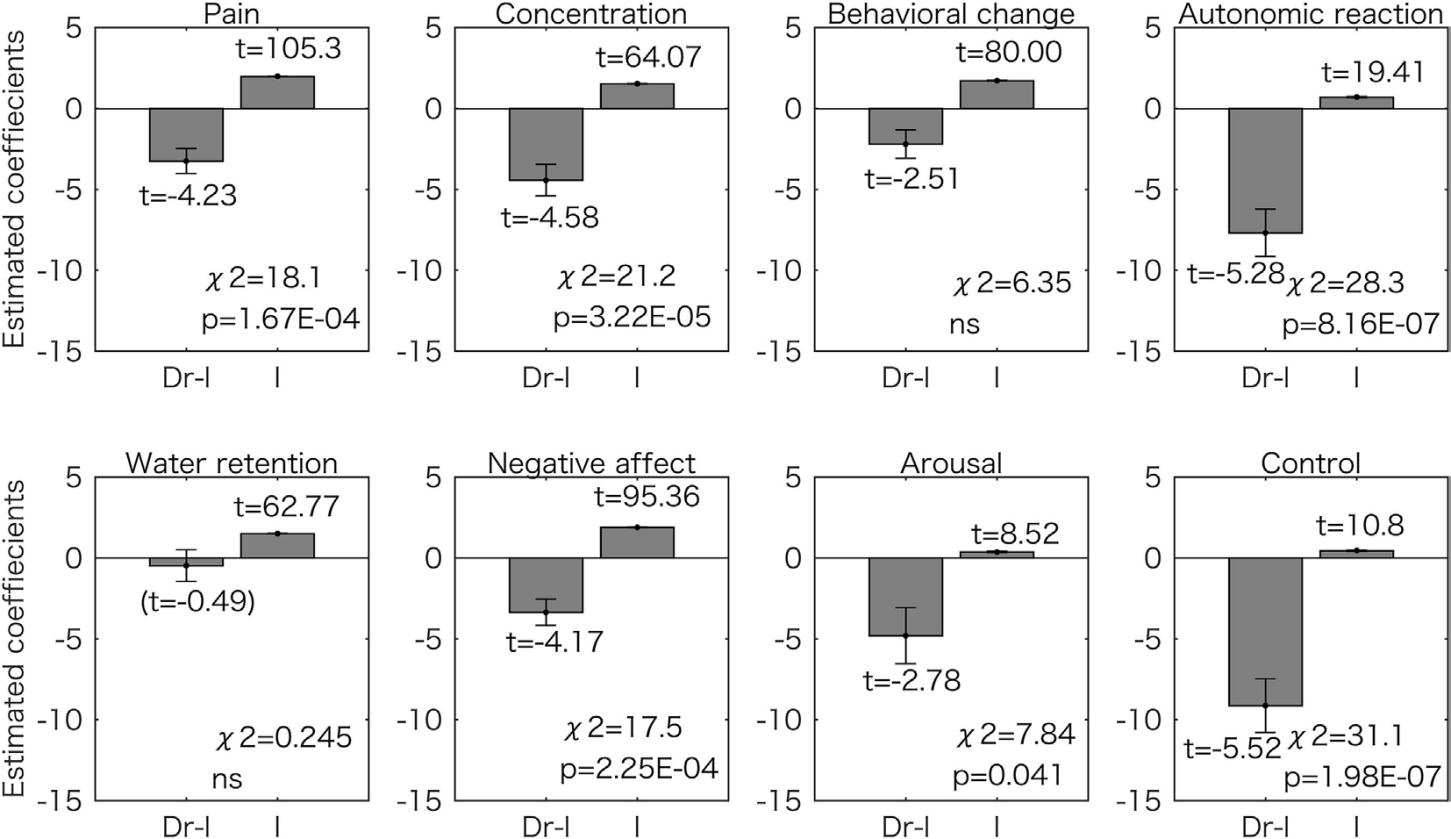
Generalized linear model analyses using one predictor for the eight symptom scores. Estimated coefficient values (±SE) are shown with *t* values. All the *t* values (except the value in the parentheses) are *p* < 0.05. The *p* values for the chi-square tests are corrected for multiple comparisons using the Bonferroni method. Dr-l was negatively related to the all scores and the models for the six symptoms were significantly different from the constant model. ns, not significant (*p* > 0.05); I, intercept.

Figure 4 shows the results for the model with one predictor (Dr-l). We found that 6 symptom scores (pain, concentration, autonomic reaction, negative affect, arousal, and control) could be predicted by Dr-l more accurately than when using the constant model (*p* < 0.05 corrected for multiple comparisons with the Bonferroni method). For all six symptoms, Dr-l was negatively related to the symptom scores.

The results for the one-predictor model using the right or left 2D:4D are shown in Table 1. The right 2D:4D was negatively related to the total scores (*p* = 2.41 × 10^−6^ by chi-square test), and the left 2D:4D was positively related to the total scores (*p* = 9.70 × 10^−6^ by chi-square test), which corresponds to the results of the two-predictor model using the data from both hands (Figure 2). The AIC from the two-predictor model was lower than that from one-predictor models, indicating that the two-predictor model was more accurate than the one-predictor models. The right 2D:4D was also negatively related to the scores of pain, concentration, autonomic reaction, and control. The left 2D:4D was positively related to the score of negative affect. The AIC from these models was higher than the corresponding symptoms’ two-predictor models. Thus, the two-predictor models were more accurate than the one-predictor models.

**Table 1:** Results of the one-predictor model using the right or left 2D:4D.

	Estimated coefficient (SE)	*t* (*p* value)	Chi^2^ (*p* value)	Akaike’s information criterion (AIC) (difference^a^)
**Total score**				
R	**−1.53 (0.33)**	**−4.72 (2.41 × 10^−6^)**	**22.2 (2.41 × 10^−6^)**	**10,198 (73)**
L	**1.47 (0.33)**	**4.42 (9.74 × 10^−6^)**	**19.6 (9.70 × 10^−6^)**	**10,201 (76)**
**Pain**				
R	**−1.49 (0.70)**	**−2.13 (0.033)**	**4.55 (0.033)**	**2,904.2 (12)**
L	1.30 (0.71)	1.81 (0.07)	3.27 (0.07)	2,905.5 (13)
**Concentration**				
R	**−2.38 (0.88)**	**−2.71 (0.0067)**	**7.34 (0.0067)**	**3,330.5 (12)**
L	1.38 (0.89)	1.55 (0.12)	2.39 (0.12)	3,335.5 (17)
**Behavioral change**				
R	−1.18 (0.80)	−1.47 (0.14)	2.15 (0.14)	2,637.3 (2)
L	0.71 (0.81)	0.87 (0.39)	0.75 (0.39)	2,638.7 (4)
**Autonomic reaction**				
R	**−4.87 (1.31)**	**−3.72 (0.0002)**	**13.9 (0.0002)**	**2,026.1 (14)**
L	1.55 (1.33)	1.16 (0.25)	1.35 (0.25)	2,038.6 (27)
**Water retention**				
R	1.03 (0.90)	1.15 (0.25)	1.33 (0.25)	2,296.6 (−1)
L	1.50 (0.91)	1.64 (0.10)	2.68 (0.10)	2,295.2 (−2)
**Negative affect**				
R	−0.62 (0.73)	−0.85 (0.40)	0.716 (0.40)	3,906.2 (17)
L	**2.29 (0.75)**	**3.06 (0.0023)**	**9.34 (0.0023)**	**3,897.6 (8)**
**Arousal**				
R	−1.90 (1.57)	−1.22 (0.22)	1.48 (0.22)	1,820.4 (4)
L	2.20 (1.60)	1.38 (0.17)	1.9 (0.17)	1,819.9 (4)
**Control**				
R	**−6.63 (1.48)**	**−4.49 (7.15 × 10^−6^)**	**20.1 (7.27 × 10^−6^)**	**2,065.2 (13)**
L	0.92 (1.51)	0.61 (0.54)	0.37 (0.54)	2,084.9 (33)

*R, right 2D:4D; L, left 2D:4D*.

*^a^The AIC for the one-predictor model minus the AIC for the two-predictor model*.

*Bold characters indicate statistically significant results*.

For all the models, we observed that the right 2D:4D was highly associated with the scores of control (estimated coefficients of −6.63 and −10.80 for the one-predictor model and two-predictor model, respectively) and autonomic reaction (the estimated coefficients of −4.87 and −8.65 for the one-predictor model and two-predictor model, respectively). Dr-l was also highly associated with the scores of control and autonomic reaction, with an estimated coefficient of −9.14, and −7.70, respectively.

## Discussion

In this study, we measured 2D:4D digit ratios in 402 young female volunteers and used an MDQ to survey their premenstrual symptoms. Digit ratio is known to be affected by both ethnicity (21) and the methods used to measure digit length (34). The mean digit ratio for the subjects in this study (0.963 ± 0.03 and 0.966 ± 0.03) corresponds to the values acquired for Japanese females in previous studies (22, 35).

The one- and two-predictors models showed that the right 2D:4D was negatively related to the total score and symptom categories of pain, concentration, autonomic reaction, and control (Figures 2 and 3; Table 1). The results suggest that the severity of such premenstrual symptoms is associated with prenatal sex hormones (testosterone and estrogen) exposure: several lines of evidence summarized by Hönekopp et al. (19) and Manning et al. (36) suggest that the human digit ratio is affected by the magnitude of prenatal sex hormone exposure and the sensitivity of androgen receptors to testosterone. Furthermore, the individual digit ratios seem to be stable (37, 38) after 2 years of age (39). A recent animal study reinforced the hypothesis that digit ratio is determined during a narrow window in early embryogenesis by the balance of testosterone and estrogen concentrations experienced by the fetus at that time (20). Thus, we conclude that the severity of a variety of premenstrual symptoms is associated to some extent with prenatal sex hormone exposure, which occurred many years before puberty. Our conclusion is supported by the observation that sex hormone concentrations do not vary with digit ratio in women of reproductive age (15). Furthermore, adult sex hormone levels (serum estrogen and progesterone levels) do not explain individual premenstrual symptoms (4).

Although it is unknown how prenatal sex hormone exposure can have significant effects on the quality of later life, several lines of relevant evidence have been reported. The age at menarche is negatively related to the right 2D:4D (40, 41), and high 2D:4D is associated with a longer reproductive period (from menarche to menopause) (42). Furthermore, women with a higher right digit ratio have been shown to have a greater number of children than women with a lower digit ratio (43). While the exact reason for these results remains unknown, the authors suggested several possibilities such as the sexual attractiveness of behavior, face, body shapes, and health status. The authors argued that facial symmetry, which is a feature of sexual attractiveness, is related to digit ratio and that symmetry in the development of the face and body may indicate developmental stability due to resistance to genetic and environmental perturbations (44). Women with a high right 2D:4D may be able to cope more easily with future dynamic fluctuations in sex hormone levels during the menstrual cycle.

Dr-l alone was found to be as a good predictor of premenstrual symptom severity as the digit ratios from both hands. The value was negatively related to the symptom scores (Figures 2 and 4). This observation corresponds to the results for the two-predictor GLM using both digit ratios: smaller right 2D:4D and larger left 2D:4D were related to a high symptom score (Figures 2 and 3). Dr-l has been shown to be negatively correlated to androgen receptor sensitivity as observed with the right 2D:4D (32). However, it is not always the case that Dr-l exhibits the same relationship with other human traits same as that reported for the right 2D:4D (16). Dr-l provide information about lateral development because the value is related to both sides of the human body.

The left 2D:4D was as accurate as the right 2D:4D for predicting premenstrual symptoms, but was positively correlated, as opposed to negatively correlated. This result is interesting considering that the left 2D:4D was significantly associated with the right 2D:4D (*r* = 0.567, *p* = 1.42 × 10^−35^). The opposite association of the left and right 2D:4D with the symptoms, however, would not be artifactually caused by the effect of multicollinearity in the two-predictor models. This is because the value of VIF (1.47) was so small to affect coefficient estimation (25). Furthermore, the one-predictor models using the left 2D:4D data showed the same tendency, that is, all the estimated coefficients had the same positive signs (Table 1). In contrast, the one-predictor model using the right 2D:4D showed that all but one estimated coefficients were negative.

The left 2D:4D may provide different information about prenatal environment than the right 2D:4D. It seems that the left 2D:4D is less affected by prenatal sex hormones than the right 2D:4D (17, 31, 36) and that the left 2D:4D is more sensitive to environmental factors other than the prenatal sex hormones (45). Recent animal studies also suggest asymmetric effects of sex hormones on both hands’ 2D:4D (20). Genetic influence was suggested to be different between the right and left 2D:4D, which are uniquely affected by the environmental factors (22). Furthermore, several studies showed that the opposite association of left and right 2D4D: left-handed writers tend to have larger left 2D:4D and a smaller right 2D:4D than right-handed writers (18). Male subjects with schizophrenia in Turkey had higher right 2D:4D and lower left 2D:4D (46). In regard to women’s quality of life, breast cancer risk was positively related to the left 2D:4D. The left 2D:4D might inform embryonic environmental factors that could cause adverse effects on the adult female quality of life in contrast to the right 2D:4D.

We report that 2 symptom scores (control and autonomic reaction) were mostly associated with 3 predictors (right and left 2D:4D and Dr-l). By contrast, 2 symptom scores (behavioral change and water retention) had no association with any of the 3 predictors (Figures 3 and 4). Although premenstrual symptoms are triggered by fluctuations in sex hormones, there is no evidence that these symptoms share a common pathophysiological origin (47). Thus, some of the symptoms may be related to the function of the central nervous system while some are related to the sensitivity of the peripheral tissues such as skeletal muscles and kidneys (47, 48). Indeed, serotonin reuptake inhibitors, which are often used to relieve severe PMS symptoms, are less effective for somatic symptoms than for mood symptoms (49, 50). Our results suggest that even the symptoms most probably related to the function of the central nervous system, such as behavioral change and control, are caused by different mechanisms.

One should be cautious in applying our results to the interpretation and prediction of PMS symptoms. We are not sure what percentage of our participants would meet the criteria for PMS because we did not diagnose whether or not the subjects suffered from PMS. Although all of our participants were university students attending classes with no apparent health problems, it is reasonable to assume that some participants would have PMS symptoms since 20–40% of women of reproductive age are diagnosed with PMS (1). It is possible, however, that the same analyses, using data derived only from women with PMS, might show different results from those reported in this study. We could not evaluate this possibility with our dataset because there was a shortage of subjects with sufficiently high scores to meet the criteria of PMS.

Several other limitations exist in this study. Premenstrual symptoms are possibly caused by the interaction of various factors including environmental and social stressors as well as hormonal changes, which we could not objectively evaluate in this study. Digit ratios are affected by various factors other than the sex hormones such as genetic influence and embryonic environmental factors other than the sex hormones (22). Furthermore, handedness may affect digit ratios (18). Although we assumed all of these as normal distribution measurement errors, they could have biased the results to some extent.

In conclusion, right- and left-hand digit ratios (2D:4D) were differentially related to the severity of premenstrual symptoms in healthy young women. Low right 2D:4D and high left 2D:4D were positively associated with high severity symptom scores. The difference between the two values (Dr-l) was negatively related to the symptom scores. When the symptoms were divided into eight categories, five categories such as pain, concentration, autonomic reaction, negative affect, and control were predicted by the two digit ratios for both hands and Dr-l, but 3 categories, behavioral change, water retention, and arousal, were not. These results suggest that the prenatal environment including sex hormones (e.g., testosterone and estrogen) exposure contributes to individual differences in the severity of premenstrual symptoms.

## Ethics Statement

This study was carried out in accordance with the Declaration of Helsinki and was approved by the Ethics Committee of Wakayama Medical University. Written informed consent was obtained from all subjects.

## Author Contributions

YK conceived and designed the experiments. TD and AI performed the experiments. TS and YK analyzed the data. YK wrote the manuscript.

## Conflict of Interest Statement

The authors declare that the research was conducted in the absence of any commercial or financial relationships that could be construed as a potential conflict of interest.
